# Revision of the *Lispe longicollis*-group (Diptera, Muscidae)

**DOI:** 10.3897/zookeys.235.3306

**Published:** 2012-10-31

**Authors:** Nikita E. Vikhrev

**Affiliations:** 1 Zoological Museum of Moscow University, Bolshaya Nikitskaya 6, Moscow, 125009, Russia (ZMUM)

**Keywords:** *Lispe longicollis* species-group, Muscidae, Diptera, key, new species, new synonym

## Abstract

The *Lispe longicollis* species-group is revised. *Lispe ethiopica* **sp. n.** is described. The following 3 new synonyms are established: *Lispe assimilis* Wiedemann, 1824 (syn: *cyrtoneurina* Stein, 1900 and *modesta* Stein, 1913); *Lispe manicata* Wiedemann, 1830 (syn: *forficata* Kurahashi & Shinonaga, 2009). Female of *Lispe microptera* Seguy, 1937 is described for the first time. Identification key for known Eurasian and African species is given.

## Introduction

The *Lispe longicollis* species-group was proposed by Hennig (1960) based on the characteristic shape of a vein M which is distinctly curved forward at apex. The species of this group also share these additional characters: *t3* with submedian *av*, *ad* and *pd* setae; abdomen with large, more or less fused trapezoid spots; frontal triangle narrow; *dc* 2+4, usually only 2 posterior pairs are strong (in *Lispe glabra* Wiedemann and *Lispe manicata* Wiedemann *dc* setae should be described as 0+2, but careful examination shows that 4 anterior pairs of minute *dc* setulae are normally present but broken in most specimens). Hennig divided the group into two subgroups.

Subgroup 1 included the species with ventral seta on *t2* and consisted of *Lispe longicollis* Meigen, 1826 (S Palaearctic) and *Lispe cilitarsis* Loew, 1856 (North Africa and Near East). In this paper another three species are added to subgroup 1: *Lispe microptera* Seguy, 1937 known from Pakistan and India and two Afrotropical species, *Lispe barbipes* Stein, 1908 and the here described *Lispe ethiopica* sp. n. The main subgroup character is the presence of fine hairs on the meron above the hind coxa; other characters: *t2* with ventral seta (except for *Lispe microptera*); male hind basitarsus curved and bears long ventral hairs (except for *Lispe longicollis*); halves of cercal plate of a subquadrate shape and strongly conjoined with each other (less so in *Lispe longicollis*); the flies inhabit banks of salted to fresh water.

Hennig’s subgroup 2 included widespread *Lispe assimilis* Wiedemann, 1824 and African *Lispe nuba* Wiedemann, 1830 which lack the ventral seta on *t2*. In this paper another three Oriental species are added to the subgroup 2: *Lispe glabra* Wiedemann, 1824, *Lispe manicata* Wiedemann, 1830 and *Lispe pacifica* Shinonaga & Pont, 1992. Subgroup 2 is characterized as follows: meron bare; *t2* without ventral seta; male hind basitarsus unmodified; halves of cercal plate of subtriangular shape and less conjoined with each other; the flies inhabit banks of fresh water only.

This revision considers Palaearctic, Afrotropical and Oriental species of the *Lispe longicollis* group. The group is absent in the Nearctic region, there are two Australian species not seen by the author, namely *Lispe weschei* Malloch, 1922 and *Lispe xenochaeta* Malloch, 1923, which also belong to the same group.

## Material and methods

The majority of the specimens studied are deposited in the Zoological Museum of Lomonosov Moscow State University, Moscow, Russia, in this case not indicated in text. Other collections are abbreviated as follows:

BMNHNatural History Museum, London, UK;


TAUITel-Aviv University, Israel;


ZINZoological Institute of the Russian Academy of Sciences, St Petersburg, Russia;


ZMHUMuseum für Naturkunde, Humboldt-Universität zu Berlin, Berlin, Germany.


The names of the collectors are abbreviated as follows: KT – Konstantin Tomkovich, NV – Nikita Vikhrev.

The following abbreviations for morphological structures are used: *f1*, *t1*, *f2*, *t2*, *f3*, *t3* = fore-, mid-, hind- femur or tibia; *ac* = acrostichal setae; *dc* = dorsocentral setae; *a*, *p*, *d*, *v* = anterior, posterior, dorsal, ventral seta(e); *prst* – presutural, *post* - postsutural.

The abbreviation for the tarsi as *tar* followed by a pair of digits separated by a hyphen was proposed by Vikhrev (2011): the first digit (1 to 3) gives the leg number and the second digit (1 to 5) the number of the tarsal segment. For example, *tar1-4* = 4th segment of fore tarsus; *tar3-1* = hind basitarsus.

Geographical coordinates are given in the Decimal Degrees format.

Synonymies are listed only for the species to which the new synonymies are considered, for full lists of synonymies see the regional Diptera Catalogues: Pont 1977, 1980 and 1986.

### Identification key to Eurasian and African species of *Lispe longicollis*-group

**Table:** 

1	Meron bare; *t2* with only 1 *pd* seta, without ventral seta. ♂: hind basitarsus not modified as below. Subgroup 2	2
–	Meron setulose above hind coxa. *t2* with ventral seta (exception *Lispe microptera*). ♂: hind basi-tarsus modified: curved and with long ventral hairs (exception *Lispe longicollis*). Subgroup 1	6
2	Disc of scutum densely brownish-grey dusted; *dc* 2+4, two last prescutellar pairs strong, others at least clearly distinct; presutural intraalar seta present; medium size species, body length 6–7.5mm	3
–	Disc of scutum mostly subshining, with a pair of thinly dusted submedian vittae only; *dc* 0+2, only last pair strong; presutural intraalar setae absent; large species, body length 8–9.5mm	5
3	♂: *f2* with setae on *av* to *pv* surfaces long and dense, the longest setae about twice as long as femur width. ♀: either *f2* in basal part with erect, rather dense setulae on *av* to *pv* surfaces, these setae at base almost equal to femoral width (*Lispe pacifica*) or *f1* ventrally with 2–3 rows of fine setulae (*Lispe nuba*)	4
–	♂: *f2* without *av*-setae and with only short *pv*-setulae which even in basal part about half as long as femur width. ♀: *f2* with only short and sparse setulae; *f1* bare on ventral surface apart from usual row of *av* setae. Africa, Palearctic and Oriental regions, Australia	*Lispe assimilis* Wiedemann
4	♂: *f1* ventrally with a dense brush of setulae placed in about 5 rows in basal half of femur and in 1–2 rows in apical half, the usual *av* row of setae on *f1* reduced to 1(2) setae at apex. *f2* with ventral setae long in basal 1/3 (2 times as long as femur width), ventral setae in median 1/3 much shorter, only as long as femur width. ♀: *f1* ventrally with 2-3 rows of fine setulae. Africa	*Lispe nuba* Wiedemann
–	♂: *f1* ventrally without setulae; a complete *av* row on *f1* present, though consists of fine setae. *f2* with ventral setae of equal length in basal 2/3 of femur, about 1.5–2 times as long as femur width. ♀: *f1* bare on ventral surface apart from usual row of *av* setae. East Asia	*Lispe pacifica* Shinonaga & Pont
5	Palpi darkened at apex. Parafacials with usual sparse fine hairs. *f*3 with submedian *av* seta long (equal to femur width) and placed beyond middle Abdominal dusted median vitta complete, although vague on anterior parts of tergites. Hind basitarsus without *v* seta at base. ♂: mid legs and wing venation modified as in Figs 15 and 16; cercal plate as shown in Fig. 11. Oriental region	*Lispe glabra* Wiedemann
–	Palpi entirely yellow. Parafacials entirely bare. *f*3 with submedian *av* seta short (half as long as femur width) and placed before middle. Abdomen with dusted median stripes conspicuous only on posterior half of tergites. Hind basitarsus with a strong “Anthomyiidae-like” *v* seta at base. ♂: mid tarsi modified as in Fig. 14; wing venation similar to females (Fig. 13); cercal plate as in Fig. 12. South of the Oriental region	*Lispe manicata* Wiedemann
6	*t2* without *av* seta (*Lispe microptera* – 1 *pd*; ♂ *Lispe barbipes* – 1 *v-pv*; ♀ *Lispe barbipes* – 1 *pd*, 1 *v-pv*)	7
–	*t2* with 1 *p*(*pd*) and 1 *av* setae	8
7	*t2* with 1 *p*(*pd*) seta. ♂ (Fig. 3): *f3* with 4–5 fine long *pv* in basal half and 1(2) *av* in basal 1/3; *tar3-1* slightly laterally compressed and outward curved, with waved ventral setulae more dense at base and at apex; *tar3-2* with waved *v* setulae; cercal plate as in Fig. 8. ♀: *f3* on *av* surface usuallywith a short *av* seta before middle (in some specimens this seta absent). Pakistan, India	*Lispe microptera* Seguy
–	*t2* with 1 *v*(*pvv*) seta. ♂ (Fig. 1): *f2* with 2-3 strong, straight and long ventral spines; *f3* in basal 1/3 with 1-2 *av* and 1 long fine *pv*; *t3* at apical 1/3 with long waved setae on *ad* to *av* surface; *tar3-1* elongated, downward curved; with waved *v* setulae. ♀: *f3* without *av* seta; *t2* with 1 *v-pv* and 1 *pd* approximated setae. Africa	*Lispe barbipes* Stein
8	*f3* with a strong submedian *av* seta. Meron with 6-8 setulae above hind coxa and 2-4 below posterior spiracle and usually with 1 setula present on katepimeron. ♂: hind tarsus simple; cercal plate as in Fig. 3. South Palaearctic from Central Europe to Transbaikalia	*Lispe longicollis* Meigen
–	*f3* without submedian *av*. Meron only with 4-5 setulae above hind coxa. ♂: hind tarsus modified	9
9	Palpi black. ♂ (Fig. 4): mid tarsus simple; *tar3-1* dorso-ventraly flattened, distinctly wider than width of *t3*; cercal plate - Fig. 5, sternite 5 – Fig. 6. Ethiopia	*Lispe ethiopica* sp. n.
–	Palpi yellow. ♂ (Fig. 2): mid tarsus with a row of curled setulae on *p* surface; *tar3-1* not widened, less wide than width of *t3*; cercal plate as in Fig. 7. Near East, N Africa	*Lispe cilitarsis* Loew

## Taxonomy

### Lispe assimilis

Wiedemann, 1824

http://species-id.net/wiki/Lispe_assimilis

Figs 10 19

Lispe quadrilineata Macquart, 1835.
Lispe incerta Malloch, 1925.
Lispe inexpectata Canzoneri & Meneghini, 1966.
Lispe cyrtoneurina Stein, 1900: 393 **syn. nov.** Type locality: Papua New Guinea, Dilo.
Lispe modesta Stein, 1913: 557 **syn. nov.** Type locality: Abyssinia, Dambelsee [= Ethiopia, Ziway Lake].

#### Material examined.

**Syntype** *Lispe modesta* Stein, 1913 ♂, (ZMHU). [**Ethiopia**] Abyssinia, Lac. Dembel [Ziway Lake], I.1912, Kovacs.

**Australia,**: ***Qld***., Townsville, 19.29°S, 146.80°E, 17.IV.2012, G.Cocks, 1♀.

**Ethiopia**: ***Amhara***, Tana Lake env., 1800m asl, 11.54°N, 37.39°E, 2–4.VIII.2012, NV, 3♂♂, 1♀; ***Oromia***, Ziway Lake, 7.91°N, 38.73°E, 12.III.2012, NV, 1♂, 1♀.

**India**: ***Goa*** state, 15.0°N, 74.1°E, 3–16.II.2008, KT, 29♂♂♀♀; ***Rajasthan*** state, Jaipur, 26.96°N, 75.85°E, 22.II.2011, NV, 7♂♂, 11♀♀; ***Uttarakhand*** state, 30.1°N, 78.2°E, 4.IX.2011, NV, 1♀.

**Israel**, Kinneret Lake env., 27.X.2011, NV, 7♂♂, 2♀♀.

[**Italy**], ***Sicilia***, Partinico L., 12.VIII.1978, S.Canzoneri, 1♀, (labeled *Lispe inexpectata*) (ZMHU).

**Myanmar**, ***Shan*** state, Inle Lake, 30.XI.2009, NV, 6♂♂, 2♀♀.

**Morocco**: ***Essaouira*** prov., Essaouira env., 27.III.2009, NV, 1♂, 3♀♀, 1–5.V.2012, NV, 1♂, 2♀♀; ***Marrakech*** prov., Marrakech, 21.III.2009, NV, 1♂, ***Tat-Tan*** prov, Draa R., 11.V.2012, NV, 1♀.

**Nigeria**, Zungeru (9.81°N, 6.16°E), 25.II.1911, J.Macfei, 1♀ (BMNH).

**Sudan**, 08.III.1929, 1♂ with Emden’s identification label *Lispe modesta* (BMNH).

**Thailand**: ***Chiang Mai*** prov., Sop Poeng env., 17.XI.2009, NV, 1♂; ***Mae Hong Son*** prov., Pai env., 19.4°N, 98.4°E, 15–25.XI.2010, NV, 14♂♂.

**Turkey**: ***Adana*** prov., Yumurtalik env., IV.2010, NV, 1♂, 1♀; ***Antalya*** prov., Manavgat env., IX.2006–9, NV, 16♂♂, 10♀♀; ***Hatay*** prov., Samandag env., IV.2010, NV, 3♂♂, 1♀; ***Mersin*** prov., Silifke env., IV.2010, NV, 1♂, 1♀; ***Sakarya*** prov., Karasu env., V.2009, NV, 1♂; ***Zonguldak*** prov., Alapli env., V.2009, NV, 1♂.

#### Distribution.

S Palaearctic, Afrotropical and Oriental regions, Australia, Oceania.

#### Synonymies.

The taxonomy of *Lispe assimilis* was considered by Shinonaga and Pont (1992). In that paper the synonymy of *Lispe quadrilineata*, *Lispe incerta* and *Lispe inexpectata* with *Lispe assimilis* was established and the related Oriental species with long ventral hairs on mid femur was described as *Lispe pacifica* Shinonaga & Pont, 1992, it was shown that *Lispe assimilis* in the sense of old authors is *Lispe pacifica*, while later authors followed this misinterpretation.

*Lispe cyrtoneurina* Stein, 1900 – syn. nov. of *Lispe assimilis*. Stein’s (1900) original description completely fits *Lispe assimilis*, the only difference found is 3 (instead of 4) *post dc*. The male lectotype of *Lispe cyrtoneurina* (stored in Genoa, Museo Civico di Storia Naturale di Genova) was reexamined by Adrian Pont. The lectotype is in poor condition, damped and mostly squashed; 4 *post dc*; everything else fit *Lispe assimilis* (Pont, pers. com. and unpublished notes).

*Lispe modesta* Stein, 1913 – syn. nov. of *Lispe assimilis*. The very short Stein’s (1913) description fits *Lispe assimilis*. Examined by me specimens from Asia and Africa were found similar, the specimens from Ziway Lake in Ethiopia are especially interesting as it is the type locality of *Lispe modesta*. In a later paper (Stein 1918: 175) Stein himself listed *Lispe assimilis* from Rangoon (Yangoon, Myanmar) as “*Lispe assimilis* Wied. var. *modesta* Stein” and wrote that the male of *Lispe assimilis* var. *modesta* (=*Lispe assimilis* in the present interpretation) differs from *Lispe assimilis* (=*Lispe pacifica* in the present interpretation) only by the absence of long ventral hairs on *f2*.

*Lispe pacifica* Shinonaga & Pont, 1992. According to the remark cited above Stein in 1918 started to regard *Lispe assimilis* and *Lispe pacifica* as variations of the same species. In fact, the separation of these species in female sex is sometimes doubtful and males have similar genitalia. Note also that in both species the pollinosity is very variable: dusting on face, parafacialia and parafrontalia from pure white to deep yellow, dusting on parafrontalia and frontal triangle from weak to strong, dusting of scutum from grey to brown, the colour of the tibiae from almost entirely yellow to almost entirely dark. I would like to report that my observations made around Pai (Thailand, Mae Hong Son province) somewhat support the valid taxonomic status of *Lispe pacifica* Shinonaga & Pont, 1992. Pai town is so far the only locality I know where both species *Lispe assimilis* and *Lispe pacifica* were found together at the same time and usually at the same pools. A series of 17 males of *Lispe pacifica* and 14 males of *Lispe assimilis* were collected. All examined males have distinct characters either of one or other species, with no intermediate specimens recorded. Thus in a sympatric condition no trace of crossbreeding between the two species has been found.

So, *Lispe assimilis* Wiedemann, 1824 = *Lispe cyrtoneurina* Stein, 1900 syn. nov. = *Lispe modesta* Stein, 1913, syn. nov.

### Lispe barbipes

Stein, 1908

http://species-id.net/wiki/Lispe_barbipes

Fig. 1

#### Material examined.

**Syntypes** 1♂, 1♀ (ZMHU). S. W. Afrika, Luderitzbucht [**Namibia**, Luderitz, 26.65°S, 15.16°E], S. Schultze 1♂; S. W. Afrika, Kalahari, Moocane, Wasserspiegel [**Botswana**, Mookane, 23.7°S, 26.6°E, water level], X.1904, S. Schultze 1♀.

As it was reported by Pont and Werner (2006): “there must be some doubt as to whether this is actually a syntype, since the locality [of ♂ syntype] was not mentioned by Stein (1908) and is on the coast of Namibia rather than at the eastern edge of the Kalahari desert in Botswana.”

**Ethiopia**, ***Afar***, Mille env., 530m asl, 11.381°N, 40.731°E, 9.VIII.2012, NV, 1♀.

**South Africa**, [***Northern Cape*** prov.], Olifantshoek [≈27.94°S, 22.74°E], 26.II.1988, D.Simon, 2 ♂♂ (TAUI).

[**Namibia**], South Africa, Van Zylserus [***Kunene*** reg., Van Zyls pass, 17.64°S, 12.71°E, 1000m asl], 12.I.1988, D.Simon, 3 ♂♂ (TAUI and ZMUM).

#### Distribution.

Afrotropical: Botswana, Ethiopia, Namibia, South Africa.

#### Redescription.

Male. Body size – 7-8 mm. Head with frontal triangle narrow. Parafacial covered with hairs. Antenna black. Arista in basal half with hairs slightly shorter than antenna width, in apical half bare. Palpus yellow. Scutum and scutellum brownish dusted with indistinct vittae, pleura brownish-grey dusted. *dc* 2+4 (strong-strong+medium-medium-strong-strong); intraalars 1+2; supraalars 1+2, the posterior one weak. Meron with setulae above hind coxa. Wing with vein R4+5 distinctly curved forward. Legs dark, but femora at apex and tibiae in basal half yellow. *f1* with a row of 6-7 strong *av* setae in apical 3/5. *t1* with a row of 7-8 short but strong *d* setae and with submedian *p* seta. *f2* with 2(3) strong, straight and long (2 times as long as femur width) ventral spines; other setae: 1(2) median *a* seta(e), 2 *p* at apex. *t2* with 1 submedian *v* seta (which is slightly shifted from true *v* position onto posterior surface and may be named “*pvv* seta”). *f3* curved; with 1-2 *av* setae and 1 long but fine *pv* in basal 1/3, *pv* preapical present, *av* preapical absent. *t3* with submedian 1 *av*, 1 *ad* and 1 *pd* setae; below middle with a row of 3-4 straight *ad*; at apical 1/3 with long waved setae on *ad* to *av* surface. Hind tarsus modified: *tar3-1* elongated, downward curved; with waved ventral setulae, these in apical 1/3 especially long; *tar3-2* thickened. Abdomen grey dusted with large lateral black spots, these on tergites 3 and 4 separated by grey vitta, on tergite 5 fused.

Females differs from male as follows: spines on *f2* absent; *t2* with 2 approximated submedian setae, shorter *p-pd* and longer *v-pv*, *f3* without *av* and *pv* setae (these characters did not mention in Stein’s (1908) original description), *t3* without long setae at apex; hind tarsus unmodified.

### Lispe cilitarsis

Loew, 1856

http://species-id.net/wiki/Lispe_cilitarsis

Figs 2 7

#### Material examined.

**Syntype** ♂, ZMHU, also seen by Hennig (1960: 426), [**Egypt**] Assyud [Asyut], Frauenfeld, 1♂.

**Egypt**: Sinai, 21.V.1981, A.Freidberg, 1♂ (TAUI); Cairo, 2♂♂, 1♀ (ZIN); Cairo, Port Said, Suez, Luxor, Aswan, 12♂♂, 6♀♀ (ZMHU).

**Ethiopia**: ***Amhara***, Tana Lake env., 1800m asl, 11.54°N, 37.39°E, 2-4.VIII.2012, NV, 2♂♂; ***Oromia***, Ziway L., 1640m asl, 7.91°N, 38.73°E, 11–13.III.2012, NV, 1♂.

**Israel**: Ma’agan Michael, 28.VII.1964, A.Valdenberg, 19♂♂, 20♀♀ (TAUI); Eilat env., 24.X.2011, NV, 10♂♂, 9♀♀.

**Morocco**: ***Tan-Tan*** prov., Draa R., 28.528°N, 10.947°W, 11.V.2012, NV, 1♂, 1♀.

#### Distribution.

Egypt, Ethiopia, Israel, Morocco. Also reliably known from Saudi Arabia and Oman (Pont 1991). In Ethiopia *Lispe cilitarsis* seems uncommon and restricted to northern regions in comparison with resembling *Lispe ethiopica* sp. nov., so specimens from Africa should be re-examined and so far I regard other Afrotropical records as doubtful.

### Lispe ethiopica

Vikhrev sp. n.

urn:lsid:zoobank.org:act:8412D7E2-3B7E-4527-A0F2-6B3DACBEB089

http://species-id.net/wiki/Lispe_ethiopica

Figs 4, 5, 6

#### Holotype:

male, Ethiopia, Oromia, Langano Lake, 1590m asl, 7.646°N, 38.706°E, 13-15.III.2012, NV (ZMUM).

Paratypes 23♂♂, 24♀♀. **Ethiopia:** Dire Dawa, Afrika, Diredaua [= Ethiopia, Dire Dawa, 9.60°N, 41.85°E], 28.X.[1945–55], O.Theodor, 1♂(TAUI); ***Oromia*:** Mojo bridge, 8.597°N, 39.111°E, 21.IX.2003, A.Freidberg, 1♂ (TAUI); Langano Lake, 1590m asl, 7.646°N, 38.706°E, 13–15.III.2012, NV, 9♂♂, 12♀♀; Ziway Lake, 1640m asl, 7.91°N, 38.73°E, 11–13.III.2012, NV, 11♂♂, 12♀♀; Abijata Lake, 1580m asl, 7.61°N, 38.65°E, 14.III.2012, NV, 1♂.

#### Description.

Male, body length 6.5–7.5 mm.

Head. Frontal triangle remarkably narrow, brownish in posterior half, yellowish-grey dusted in anterior half. Interfrontalia blackish-brown. Fronto-orbital plate blakish-brown in posterior third, yellowish-grey dusted anteriorly; with 3-5 inclinate and 2 proclinate setae and dense hairs in outer row. Parafacial and cheek whitish dusted, occiput grey, parafacial with a row of hairs. Antenna black, postpedicel short, only 2 times longer than pedicel. Arista with hairs half as long as antenna width. Vibrissae medium strong. Palpi blackish.

Thorax. Pleura densely grey dusted, scutellum and disc of scutum brown, thinly dusted, with a pair of densely dusted prescutellar ochrous spots; vittae indistinct. Presutural *ac* in 4 irregular rows; *dc* 2+4, four anterior pair medium strong, two posterior pairs strong; intraalars 1+2; supraalars 1+2; katepisternals 1+2; anepimeron with 11-13 setulae; meron with 3-5 setulae above hind coxa. Wings hyaline, slightly brownish, vein Mdistinctly curved forward at apex, calypters white, halter yellow.

Legs black with grey dusting, but knees and base of tibiae yellowish. *f1* with a row of *pd* setae and a row of *pv* setulae; *t1* with submedian *p* seta. *f2* with *a* seta at middle and 2 *pd* preapicals; *t2* with *p* seta at middle and *av* seta in apical third; mid tarsus simple. *f3* with 1-2 fine *v* setulae at base, at apex with 1 short *av* and 1 short *pv*; *t3* with submedian *ad* and *pd* setae and with long fine *av* at apical third, setulae in the *ad* row elongated. Hind tarsus modified: *tar3-1* dorso-ventrally flattened, distinctly wider than width of *t3*, on *av* surface with a dense row of fine curled setulae.

Abdomen with dense whitish dusting; tergites 3 to 5 with a pair of large black fused spots each. Cercal plate and sternite 5 as in Figs 5 and 6.

Female differs from male as follows: body length 7-8 mm; *t3* with *av* seta strong; hind tarsus simple.

#### Diagnosis.

*Lispe ethiopica* sp.n. is related to *Lispe cilitarsis* Loew, 1856 and probably was overlooked due to that resemblance. These two species may be reliably distinguished in both sexes as recommended in the identification key above.

**Etymology**. Named after the locality of the type series.

### Lispe glabra

Wiedemann, 1824

http://species-id.net/wiki/Lispe_glabra

Figs 11 15, 16

#### Material examined.

**India**, ***Goa*** state, 15.0°N, 74.1°E, 3–16.II.2008, KT, 3♂, 7♀♀.

**Myanmar**, ***Shan*** state, Inle L., 30.XI.2009, NV, 3♀♀.

**Thailand**: ***Chanthaburi*** prov., Khao Khitchakut env., 12.82°N, 102.13°E, XI.2009, NV, 3♀♀; ***Chonburi*** prov., Pattaya env., XII.2008–9, NV, 40 ♂♂, ♀♀; ***Mae Hong Son*** prov., Pai env., 11.XI.2009, NV, 5♀♀; ***Phuket*** prov., Nai Thon beach, 20.II.2009. NV 3♂♂, 4♀♀, NV; ***Phang Nga*** prov., Thai Mueang env., 18.II.2009, NV, 1♂, 6♀♀; ***Rayong*** prov., Ban Phe env., 12.64°N, 101.46°E, NV, 3♀♀.

#### Distribution.

Oriental region.

#### Descriptive notes.

Body length 8.5–9.5 mm. Wings slightly brownish infuscated. Vein *M* gradually curved forward from level of crossvein *dm-cu*, cell *r*4+5 is almost closed and distance between veins M and R4+5 at wing margin is shorter than crossvein *rm*. Vein CuA2 not reaching wing margin, extending only to crossvein *dm-cu*; crossvein *dm-cu* skewed, it reaches vein *M* at acute angle of about 45˚. There is a downcurved fold surrounded by long microtrichia along posterior margin of wing between veins M and A2, microtrichia directed outward to the fold. Mid legs: *f*2 with remarkable row of very dense curled Velcro fastener-like setae in *pv* position in basal 2/3; *t*2 in apical 1/4 with a row of long ventral hairs; *tar2-1* with a complete row of long curved *pv* setulae. Male is unmistakable due to modified wings and mid legs. Female differs from female of *Lispe manicata* as given in the key.

### Lispe longicollis

Meigen 1826

http://species-id.net/wiki/Lispe_longicollis

Fig. 9

#### Material examined.

[**Iran**], Sistan [***Sistan and Baluchestan*** prov., ≈27°N, 61°E], 19–21.V.1898, Zarudniy, 2♂♂ (ZMHU).

**Hungary**, Kalocsa, [46.5°N, 19.0°E] , Thalhammer, 2♀♀ (ZMHU).

**Kazakhstan**: ***Atyrau*** reg., Atyrau env., 47.0N, 51.8E, 21.V.2011, KT, 21♂♂, 17♀♀; ***Kyzylorda*** prov., Syr Darya R., KT, 26 ♂♂, ♀♀, ***Lispe Kazakhstan*** reg., Uralsk env., 51.07°N, 51.05°E, 26.VIII.2012, KT, 6♂♂, 7♀♀.

**Russia: *Astrakhan*** reg., Baskunchak L., 48.19°N, 46.82°E, 2–4.V.2010, KT, 7♂♂, 2♀♀; ***Gorno-Altay*** reg., Ust-Koksa env., 50.26°N, 85.61°E, 25.VI.2007, O.Kosterin, 1♀; ***Kalmykia*** reg., 47.875°N, 44.601°E, 08.VI.2012, NV, 3♂♂, 1♀; ***Khakassia*** reg., Shira env. 54.422°N, 90.147°E, 26.VI.2011, KT, 2♀♀; ***Krasnodar*** reg., Pshada R., 44.39°N, 38.34°E, 6.IX.2009, KT, 6♂♂, 3♀♀; ***Omsk*** reg., Omsk, 54.97°N, 73.36°E, 15.VI.2011, O.Kosterin, 1♀; ***Orenburg*** reg., Sol-Iletsk env., 51.342°N, 55.013°E, 28.VIII. KT, 7♂♂, 7♀♀; ***Saratov*** reg.,Saratov env., 51.60°N, 46.35°E, 24.VIII.2012, KT, 1♂, 4♀♀; ***Stavropol*** reg.,saltish pond, 45.245°N, 42.665°E, 09.VI.2012, NV, 4♂♂, 2♀♀; ***Volgograd*** reg., 50.418°N, 42.760°E, 7.VI.2012, NV, 1♂, Sarpa L., 48.35°N, 44.61°E, 7.VI.2012, NV, 3♂♂, 2♀♀; ***Zabaykalsky*** reg., Zun-Torey soda Lake, 50.01°N, 115.72°E, 30.VII.2011, A.Medvedev, 1♂.

[**Slovenia**], Illyria, Gorz [Gorica, ≈ 45.9°N, 13.7°E], IX.67, Mik, 1♂ (ZMHU).

**Tajikistan**, ***Khatlon*** prov.; Jilikul env. (37.5°N, 68.5°E), 16.V.1987, M.Krivosheina, 2♂♂, 2♀♀; **Turkey:** ***Antalya*** prov., Manavgat env., 36.76°N, 31.45°E, X.2006–7, NV, 1♂, 2♀♀; ***Hatay*** prov., Samandag env., 36.07°N, 35.96°E, 16.IV.2010, NV, 1♂; ***Kayseri*** prov., Subashi env., 38.51°N, 35.19°E , 19.IV.2010, NV, 1♀; ***Konya*** prov., Beyshehir Lake, 37.79°N, 31.64°E, 11.IX.2009, NV, 18 ♂♂, ♀♀; ***Mersin*** prov., Silifke env., 36.31°N, 34.02°E, 22.IV.2010, NV, 2♂♂, 8♀♀.

**Turkmenistan**: ***Balkan*** prov., Atrek R., (37.7°N, 54.8°E), 28.VII.1932, Ushinsky, 1♀; ***Mary*** prov.; Badhyz NR (35.7°N, 61.8°E), V-VI.1991, A.Ozerov, 1♂, 6♀♀.

**Ukraine**, ***Donetsk*** reg., Volnovakha distr, 47.51°N, 37.68°E, 25.VIII.2008, KT, 1♂, 2♀♀;

**Uzbekistan**, ***Karakalpakstan*** reg., Muynak env. (43.76°N, 59.03°E), VI.1957, V.Sychevskaya, 3♂♂, 9♀♀.

**Distribution**. Southern Palaearctic, from 13°E to 116°E; from 55°N to 35°N.

### Lispe manicata

Wiedemann, 1830

http://species-id.net/wiki/Lispe_manicata

Figs 12 13, 14

Xenolispa chiragrica Séguy, 1948.
Lispe forficata Kurahashi & Shinonaga, 2009: 303 syn. nov. Type locality: Malaysia, Borneo, Sarawak, Bario [3.75°N, 115.45°E].

#### Material examined.

[**Indonesia**], [***Java***], Batavia, VI.1908, Jacobson, 1♀ (ZMHU).

**Cambodia**, ***Sianoukville*** prov., Ream Nat. Park, 10. 516°N, 103.617°E, 20.IV.2010, O.Kosterin, 1♂.

**Thailand**, ***Phuket*** prov., 08.043°N, 98.277°E, 21–26.II.2009, NV, 3♂♂, 2♀♀.

#### Distribution.

South-East Asia: Cambodia, Malaysia (Borneo), Indonesia (Java), Singapore (Séguy 1948), S Thailand, S Vietnam (Séguy 1948).

#### Synonymy.

The illustrations of wonderfully modified male mid tarsi and characteristic genitalia given by Kurahashi and Shinonaga (2009: fig. 1 c–d, 3 a–d) for *Lispe forficata* suggest it’s conspecifity with *Lispe manicata*. So, *Lispe manicata* Wiedemann, 1830 = *Lispe forficata* Kurahashi & Shinonaga, 2009, syn. n.

The characters of female of *Lispe forficata* were shortly mentioned by Kurahashi and Shinonaga (2009), but these authors did not compare *Lispe forficata* with the most related species *Lispe glabra* and, in my opinion, the diagnostically important characters were either not mentioned or given erroneously. Therefore I find it necessary to provide the description of the female below.

#### Description of female.

Body length 8.5–9.5 mm.

Head. Interfrontalia matt black; frontal triangle brownish-black, subshining, narrow, reaching to lunula. Upper half of fronto-orbital plates brownish-black, subshining, lower part dirty-golden dusted, 3-4 inclinate, 2 reclinate setae and a dense outer row of setulae. Parafacials densely whitish dusted, entirely bare. Gena whitish dusted, about 1.5 times as wide as postpedicel; occiput grey dusted, but less dusted in upper 1/3. Vibrissae strong. Antennae black, long; arista haired on basal half or slightly more, longest aristal hairs half as long as width of antenna. Palpi entirely yellow.

Thorax. Pleura and dorsolateral area, including postpronotal lobe and notopleuron, densely grey dusted. Disc of scutum mostly subshining brownish-black, with a pair of thinly dusted vittae situated mesad to *dc* rows, disc of scutellum subshiny brownish-black. *ac* hairs in 5-6 irregular rows; *dc* 0+2, only posterior pair strong (hair-like and indistinct posterior *prst dc* and 1-2 pairs anterior *post dc* may be found in some specimens); intraalars 0+1, presutural one absent; supraalars 1+2, the posterior one weak. Katepisternal setae 1+2; anepimeron with 9 (8-10) fine hairs, meron and katepimerom bare. Scutellum bare below at apex. Mesothoracic spiracle yellowish. Wings hyaline, slightly brownish infuscated, vein Mdistinctly curved forward at apex, calypters whitish, halteres yellow.

Legs. Legs long, densely grey dusted; black including coxae, but knees and basal half of *t2* and *t3* brownish-yellow; *f*1 with a full row of 6 (5-7) *pd* setae and with a row of fine *pv* setulae, but in apical half 2-4 setae in *pv* row are strong (longer than tibial diameter, stronger than setae in *pd* row); *t1* with *p* seta, preapical *d* and apical *pd* and *pv*; *f*2 thickened in basal half, *f*2 with row of 3-5 *a*-setae in basal 1/3 and 2 *pd* at apex; *t*2 with submedian *pd*; *f*3 with short *av* before middle and shorter than setae of *ad* row, with a full row of *ad* subequal to femur depth, preapicals: *av* and *pv*; *t*3 with submedian *av*, *ad* and *pd* setae; hind basitarsus with *av* seta at base.

Abdomen: black with grey pollen. Tergites 1+2 to 5 each with a large blackish spots on dorsal and lateral sides, these spots on tergites 3 and 4 divided at midline by grey dusted interrupted vitta.

Male. Similar to female, but mid tarsi modified: apex of mid basitarsus and 4 apical tarsal segments bright yellow; *tar2-2* and *tar2-3* enlarged, *tar2-4* and *tar2-5* strongly enlarged; apex of *tar2-1* and *tar2-2* with long *pd-p* setae. Cercal plate as shown on Fig. 12.

### Lispe microptera

Séguy, 1937

http://species-id.net/wiki/Lispe_microptera

Figs 3 8

#### Material examined.

**India**: ***Rajasthan*** state: Jaipur, 26.96°N, 75.85°E, 21–22.II.2011, NV, 10♂♂, 7♀♀; Sambhar salt-lake, 26.916°N, 75.190°E, 23.II.2011, NV, 8♂♂.

#### Distribution.

India, Rajasthan and Pakistan, Karachi (type locality).

#### Description of female.

Body length 7–7.5 mm, wing length 6mm.

Frontal triangle narrow, yellowish dusted; interfrontalia brownish-black. Fronto-orbital plate blackish grey dusted, with 4 inclinate and 2 proclinate setae and dense hairs in outer row. Parafacial covered with hairs. Antenna black, postpedicel short. Arista with hairs two times shorter than antenna width, in apical third bare. Palpus narrow, dirty-yellow.

Thorax. Scutum and scutellum brownish dusted with a pair of indistinct vittae, pleura grey dusted.

*dc* 2+4 (medium - medium + medium/weak-medium/weak-strong-strong). Meron with 3-4 setulae above hind coxa, anepimerom with about 15 setulae. Wing with vein R4+5 distinctly curved forward.

Legs. Femora dark with yellow apex, tibiae yellow in basal half and dark in apical half, tarsi black.

*f1* with a complete row of 10-12 *pv* setae. *t1* with submedian *p* seta. *f2* with a row of short *a* setae in basal half and with 2 *pd* at apex. *t2* with 1 submedian *p* seta. *f3* slightly curved; with a short *av* seta at basal 1/3 (absent in some specimens) and short *pv* at apex, *av* preapical absent. *t3* with 1 *ad* and 1 *pd* setae at middle. Hind tarsus unmodified.

Abdomen grey dusted with large dorsal black spots separated by anteriorly interrupted grey vitta.

Male differs from female as follows: body length 6.5-7 mm, wing length 5-5.5 mm; *f2* with a complete row of fine *v* setae about as long as femur width; *f3* in basal half with 4–5 fine long (2–2.5 femur width) *pv* setae and 1(2) *av* in basal 1/3; hind tarsus modified: *tar3-1* slightly laterally compressed and outward curved, with waved ventral setulae more dense at base and at apex; *tar3-2* with waved ventral setulae; male cercal plate as in Fig. 8.

### Lispe nuba

Wiedemann, 1830

http://species-id.net/wiki/Lispe_nuba

#### Material examined:

**Ethiopia**: ***Amhara*:** Tana Lake env., 1800m asl, 11.54°N, 37.39°E, 2–4.VIII.2012, NV, 6♂♂, 7♀♀; Hayk L., 1920m asl, 11.325°N, 39.688°E, 06.VIII.2012, NV, 3♂♂, 2♀♀; Karakore env., 1500m asl, 10.375°N, 39.933°E, 08.VIII.2012, NV, 4♂♂, 1♀; ***Oromia*:** Dedre Zeit, Hora L., 1900m asl, 8.757°N, 38.993°E, 10.VII.2012, NV, 3♂♂, 3♀♀.

**Egypt**: Cairo, 5♂♂, 1♀, with Becker, Kowarz and Hennig determination labels (ZIN).

**Israel**: Yeruham (30.99°N, 34.90°E), 22.VII.1962, J.Kugler, 1♂, 1♀ (TAUI).

#### Distribution.

Africa and Near East.

#### Remarks.

Emden (1941) in the key to African *Lispe* wrote that in *Lispe nuba* “front tibiae without a *pv*”. It is not correct, all examined specimens have *t1* with *pv* seta in both sexes, though short in males.

### Lispe pacifica

Shinonaga & Pont, 1992

http://species-id.net/wiki/Lispe_pacifica

Figs 17, 18

#### Material examined.

**Cambodia**, ***Koh Kong*** prov., 11.605°N, 103.046°E, XII.2010, NV, 2♀♀.

[**Taiwan**] Formosa: Takao, [22.6N 120.3E], 7.VII.1907, H.Sauter, 6♂♂, 6♀♀, Anping, [23.0N, 120.2E], IX.1908, H.Sauter, 7♂♂, 5♀♀ (ZMHU).

**Thailand**: ***Chanthaburi*** prov., Khao Khitchakut env., XI.2009, NV, 1♀; ***Chiang Mai*** prov., Sop Poeng env., XI.2009, NV, 1♂; ***Chonburi*** prov., Pattaya env., XI.2007–XII.2009, NV, 28 ♂♂, ♀♀; ***Mae Hong Son*** prov., Pai env., 19.4°N, 98.4°E, 15–25.XI.2010, NV, 19♂♂; ***Nakhon Ratchasima*** prov., Khao Yai NP; II.2009; NV, 1♂, 1♀; ***Phang Nga*** prov., Khao Lak env., XII.2010, NV, 3♂♂, 1♀; ***Phuket*** prov., Nai Thon beach, II.2009; NV, 4♂♂, 1♀; ***Rayon*g** prov., Ban Phe env., XI.2009, NV, 2♂♂, 2♀♀; ***Sa Kaew*** prov., Mueang Sa Kaeo, II.2009; NV, 1♂.

#### Distribution.

Widespread in South-East Asia.

**Figure 1–3.**
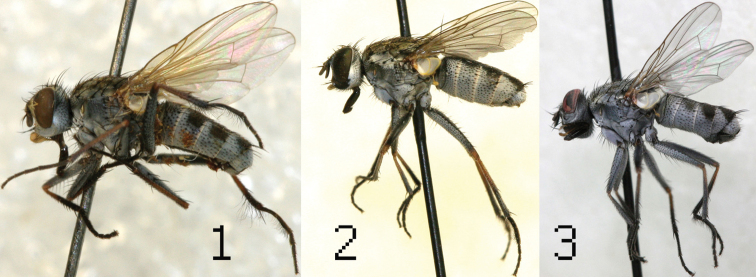
♂♂ *Lispe barbipes* Stein (**1**) *Lispe cilitarsis* Loew (**2**) *Lispe microptera* Séguy (**3**).

**Figure 4–6.**
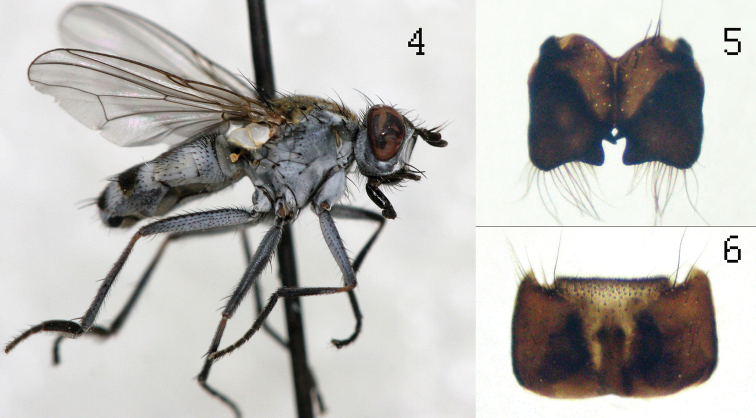
*Lispe ethiopica* sp.n.: male Holotype (**4**) cercal plate (**5**) sternite 5 (**6**).

**Figure 7–9.**
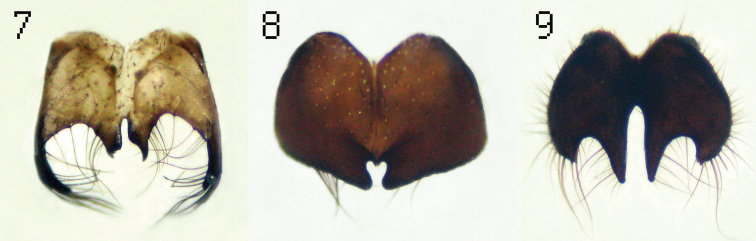
Cercal plates: *Lispe cilitarsis* Loew (**7**) *Lispe microptera* Séguy (**8**) and *Lispe longicollis* Meigen (**9**).

**Figure 10–12.**
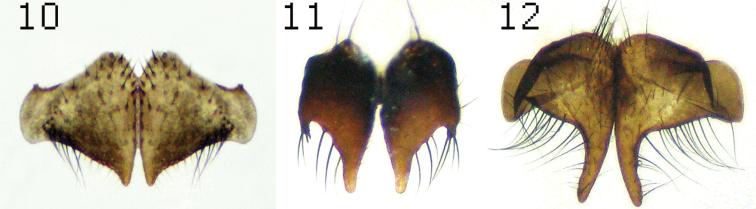
Cercal plates: *Lispe assimilis* (**10**) *Lispe glabra* Wiedemann (**11**) and *Lispe manicata* Wiedemann (**12**).

**Figure 13–16.**
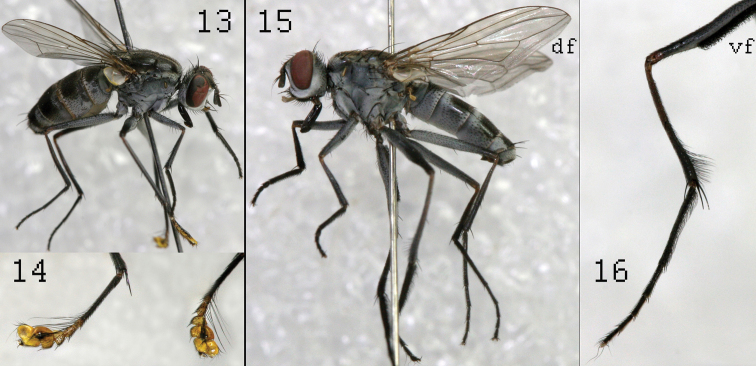
*Lispe manicata* Wiedemann: male (**13**) male mid tarsi (**14**) *Lispe glabra* Wiedemann: male (**15**) male mid tarsus (**16**), **df** – downcurved fold; **vf** – “velcro fastener- like setae.

## Ecology

As it was mentioned above, the species of the subgroup 1 of the *Lispe longicollis* group may be found both on the freshwater and salted basins. *Lispe ethiopica* sp.n. was equally common on the freshwater Ziway Lake, on the brackish (2g/l) Langano Lake and on the salt (26g/l) Abijata Lake. *Lispe microptera* was collected at brackish lakes and ponds around Jaipur and on the hypersaline (70-300g/l depending on the season) Sambhar Lake. *Lispe longicollis* was collected in spring time on the hypersaline Baskunchak Lake, on the seashore salted marshes in Mersin province of Turkey and on the freshwater Titreyen Lake in Antalya province. *Lispe cilitarsis* in Israel near Eilat was also found at freshwater of cattle drinking bowl and on a hypersaline lake shore, although in the latter case *Lispe cilitarsis* avoided the sites covered with dry salt where only *Lispe halophora* Becker, 1903 was still present.

In contrast to this salt-tolerance, the species of the subgroup 2 of the *Lispe longicollis* group were observed on freshwater basins only: river banks, rice fields or freshwater lakes/ponds. All species but one prefer open sites, *Lispe manicata* seems to be the species of the forest rivers and streams where it was collected in Thailand and Cambodia, the same natural habitat was reported by Kurahashi and Shinonaga (2009) (for *Lispe forficata*) in Malaysian Borneo.

**Figure 17–19.**
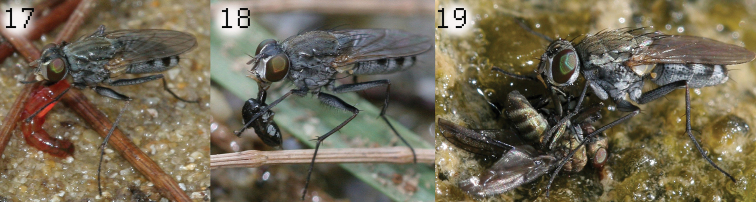
Feeding. Thailand, Phuket: male *Lispe pacifica*: with Chironomidae larva (**17**) and with dead spider (**18**) Turkey, Antalya, female *Lispe assimilis* with prey - *Paracoenia fumosa* (Ephydridae) (**19**).

The species of the *Lispe longicollis* group mostly feed on slow moving living prey like Nematocera larvae (Fig. 17), but also were observed feeding on dead arthropods (Fig. 18) or even successfully hunting on a small Diptera imago like *Paracoenia*, Ephydridae (Fig. 19) or *Syntormon*, Dolichopodidae.

## Correction

I have to apologize for an unfortunate mistake in my previous paper on *Lispe* taxonomy (Vikhrev 2011, fig. 2): sternite 5 of *Lispe draperi* Séguy, 1933 was attributed to *Lispe tentaculata* (De Geer, 1776) and vice versa.

## Supplementary Material

XML Treatment for Lispe assimilis

XML Treatment for Lispe barbipes

XML Treatment for Lispe cilitarsis

XML Treatment for Lispe ethiopica

XML Treatment for Lispe glabra

XML Treatment for Lispe longicollis

XML Treatment for Lispe manicata

XML Treatment for Lispe microptera

XML Treatment for Lispe nuba

XML Treatment for Lispe pacifica

## References

[B1] Emden FI van (1941) Keys to the Muscidae of the Ethiopian region: Scatophaginae, Anthomyiinae, Lispinae, Fanniinae. Bulletin of Entomological Research 32: 251-275. doi: 10.1017/S0007485300017193

[B2] Hennig W (1960) Family Muscidae (Lieferung 209 and 213). In: Lindner E (Ed.) Die Fliegen der Palaarktischen Region. Stuttgart 63b, 385–480.

[B3] Kurahashi H Shinonaga S (2009) Two new species of the Muscid flies from Sarawak, East Malaysia (Diptera, Muscidae). Japanese Journal of Systematic Entomology 15(2): 299–306.

[B4] Pont AC (1977) Family Muscidae. In: Delfinado MD Hardy DE (Eds) Catalogue of the Diptera of the Oriental Region 3. University Press of Hawaii, Honolulu, 451–523.

[B5] Pont AC (1980) Family Muscidae. In: Crosskey RW (Ed.) Catalogue of the Diptera of the Afrotropical Region. British Museum (Natural History), London, 721–761.

[B6] Pont AC (1986) Family Muscidae. In: Soós A Papp L (Eds) Catalogue of Palaearctic Diptera 11. Akadémia Kiadó, Budapest, 57–215.

[B7] Pont AC (1991) A review of the Fanniidae and Muscidae of the Arabian Peninsula. Fauna of Saudi Arabia 12: 312-365.

[B8] Pont AC Werner D (2006) The Types of Fanniidae and Muscidae (Diptera) in the Museum für Naturkunde, Humboldt-Universität zu Berlin, Germany. Mitteilungen aus dem Museum für Naturkunde in Berlin – Zoologische Reihe 82(1): 3–139. doi: 10.1002/mmnz.200600001

[B9] Séguy E (1948) Dipteres nouveaux ou peu connus d’extreme-Orient. Notes d’Entomologie Chinoise 12(14): 153–172.

[B10] Shinonaga S Pont AC (1992) A new species of the genus *Lispe* Latreille, with notes on two related species, L. assimilis Wiedemann and *L. microptera* Séguy (Diptera, Muscidae). Japanese journal of entomology 60 (4): 715-722.

[B11] Stein P (1900) Einige dem Genueser Museum gehorige aus Neu-Guinea und Umgegend stammende Anthomyiden. Annali del Museo Civico di Storia Naturale di Genova 40: 374-95.

[B12] Stein P (1908) Zoologische und anthropologische Ergebnisse einer Forschungsreise im westlichen und zentralen Sudafrika ausgefuhrt in den Jahren 1903–1905 mit Unterstutzung der Kgl. Preussischen Akademie der Wissenschaften zu Berlin von Dr. Leonhard Schultze, Professor der Erdkunde an der Universitat Jena. Erster Band: Systematik und Tiergeographie. IV. Insecta (Erste Serie). D. Diptera. 4. Anthomyidae. – Denkschriften der medizinisch-naturwissenschaftlichen Gesellschaft zu Jena 13: 171–174.

[B13] Stein P (1913) Neue afrikanische Anthomyiden. Annales historico-naturales Musei nationalis Hungarici 11: 457-583

[B14] Stein P (1918) Zur weitern Kenntnis aussereuropaeischer Anthomyiden. Annales Historico-Naturales Musei Nationalis Hungarici 16: 147-244.

[B15] Vikhrev N (2011) Review of the Palaearctic members of the *Lispe tentaculata* species-group (Diptera, Muscidae): revised key, synonymy and notes on ecology. ZooKeys 84: 59-70. doi: 10.3897/zookeys.84.819 PMC3088069 21594166

